# Marble melancholy: using crossmodal correspondences of shapes, materials, and music to predict music-induced emotions

**DOI:** 10.3389/fpsyg.2023.1168258

**Published:** 2023-08-31

**Authors:** Bruno Mesz, Sebastián Tedesco, Felipe Reinoso-Carvalho, Enrique Ter Horst, German Molina, Laura H. Gunn, Mats B. Küssner

**Affiliations:** ^1^Instituto de Investigación en Arte y Cultura, Universidad Nacional de Tres de Febrero, Sáenz Peña, Argentina; ^2^Programa de Investigación STSEAS, EUdA, UNQ, Bernal, Argentina; ^3^Universidad de los Andes School of Management, Bogotá, Colombia; ^4^Bayesian Solutions LLC, Charlotte, NC, United States; ^5^Department of Public Health Sciences, University of North Carolina at Charlotte, Charlotte, NC, United States; ^6^School of Data Science, University of North Carolina at Charlotte, Charlotte, NC, United States; ^7^Faculty of Medicine, Department of Primary Care and Public Health, Imperial College London, London, United Kingdom; ^8^Department of Musicology and Media Studies, Humboldt-Universität zu Berlin, Berlin, Germany

**Keywords:** crossmodal correspondences, music-induced emotions, shapes, materials, machine learning, random forests, sensory interactions

## Abstract

**Introduction:**

Music is known to elicit strong emotions in listeners, and, if primed appropriately, can give rise to specific and observable crossmodal correspondences. This study aimed to assess two primary objectives: (1) identifying crossmodal correspondences emerging from music-induced emotions, and (2) examining the predictability of music-induced emotions based on the association of music with visual shapes and materials.

**Methods:**

To achieve this, 176 participants were asked to associate visual shapes and materials with the emotion classes of the Geneva Music-Induced Affect Checklist scale (GEMIAC) elicited by a set of musical excerpts in an online experiment.

**Results:**

Our findings reveal that music-induced emotions and their underlying core affect (i.e., valence and arousal) can be accurately predicted by the joint information of musical excerpt and features of visual shapes and materials associated with these music-induced emotions. Interestingly, valence and arousal induced by music have higher predictability than discrete GEMIAC emotions.

**Discussion:**

These results demonstrate the relevance of crossmodal correspondences in studying music-induced emotions. The potential applications of these findings in the fields of sensory interactions design, multisensory experiences and art, as well as digital and sensory marketing are briefly discussed.

## 1. Introduction

Crossmodal correspondences have been defined as the ability to map or associate features across different sensory modalities (Spence, 2011; Spence and Parise, 2012). In the auditory domain, crossmodal correspondences between pitch and visual or spatial features have been a recurrent topic of studies. For instance, most people match high-pitched sounds with small, bright objects located high up in space (Spence, 2011). However, there is also evidence for stable mappings between pitch and other sensory modalities such as taste and smell (Ward et al., 2022). In a study by Crisinel and Spence (2012), high-pitched sounds were associated with sweet and sour tastes, while low-pitched sounds were preferably matched with umami and bitter tastes. Belkin et al. (1997) demonstrated matches between certain auditory pitches with specific odorants according to their odor quality; just as specific odors are matched to certain types of instruments (Crisinel and Spence, 2012), or basic tastes to colors and shapes (Turoman et al., 2018; Lee and Spence, 2022). Turning to musical stimuli, Palmer et al. (2013) found music-color correspondences using classical orchestral pieces. In a recent study, Albertazzi et al. (2020) even found robust crossmodal associations between paintings by Kandinsky and music by Schönberg (for a narrative historical review of crossmodal correspondences between color and sound, see Spence and Di Stefano, 2022).

Spence (2011) proposed three different types of crossmodal correspondences: structural, statistical, and semantic. Structural correspondences are based on common neural encoding across the senses. Statistical correspondences come from the statistical regularities of the multisensory environment such as, for example, the physical correlation between pitch and size. Semantic correspondences are based on a common vocabulary describing stimuli in different sensory modalities, as in the use of “sweet” to describe music and taste (Mesz et al., 2011). Spence (2011) further points out that crossmodal correspondences between features of stimuli can also be established based on the emotional effects these stimuli have on an observer. Such emotionally-mediated correspondences are thought to be based on the matching of similar emotions or hedonic valence related to each of the associated stimuli (Palmer et al., 2013).

Stimuli that often give rise to (strong) emotional responses such as music are therefore likely to give rise to emotionally-mediated crossmodal correspondences. In fact, music-color and music-painting correspondences can often be predicted by the emotional ratings of the stimuli involved (Spence, 2020). And color has been shown to influence music-induced emotions. For instance, in a study by Hauck et al. (2022), judgments on the emotional impact of musical pieces changed in accordance to the emotions attributed to colored lighting. This mechanism of “emotion transfer” has been found also between other senses. For example, the experience of drinking coffee while listening to music was largely determined by the emotional effect of the music that was playing at that moment (Galmarini et al., 2021). Another study showed that pleasant sounds enhanced odor pleasantness (Seo and Hummel, 2011). Below, we review previous findings on musical emotions, the emotional responses elicited by visual shapes and materials and their crossmodal correspondences with music features, and introduce the research hypotheses that shaped the rationale of the present work.

### 1.1. Theoretical framework

Our aim was to investigate crossmodal correspondences between (a) music-induced emotions and freely drawn visual shapes representing those emotions, and between (b) music-induced emotions and materials associated with them (such as those employed, for instance, in concert halls, furniture, or present in natural environments). A further aim was to predict music-induced emotions from musical excerpts, visual shapes, and materials matched with those emotions.

#### 1.1.1. Music-induced emotions

Music can arouse a wide range of powerful emotions in a listener (Juslin, 2019), and two well-known scales have been developed in order to discretely model music-induced emotions. GEMS – The Geneva Emotional Musical Scale - is a model and instrument specifically designed to capture emotions evoked by music (Trost et al., 2012). GEMS comprises nine categories of musical emotions (wonder, transcendence, tenderness, nostalgia, peacefulness, energy, joyful activation, tension, and sadness). More recently, Coutinho and Scherer (2017) introduced the GEneva Music-Induced Affect Checklist (GEMIAC), as a brief instrument for analysis of music-induced emotions. GEMIAC was designed to extend and complement the GEMS, across intensity and affective responses to music.

Several researchers also propose a more parsimonious model for music-induced emotions, suggesting that music essentially communicates two dimensions of core affect: valence and arousal (Eerola and Vuoskoski, 2011; Flaig and Large, 2014; Cespedes-Guevara and Eerola, 2018). Both of these dimensions have emerged during experiments involving crossmodal correspondences between music and visual features (Palmer et al., 2013), as well as correspondences of music with other sensory modalities, for example between music and taste/flavor (Reinoso-Carvalho et al., 2019, 2020; Motoki et al., 2022). Importantly, valence and arousal (or activity) also represent two core dimensions of Osgood’s semantic differential technique. Osgood et al. (1957) showed that most variation in individuals’ connotative meanings of aesthetic stimuli can be explained by three dimensions: valence, activity, and potency. However, crossmodal correspondences between music and other (sensory) stimuli (e.g., colors or paintings) can often be accounted for by emotional mediation of specific emotions associated with both music and visuals (for a more detailed discussion, see Spence, 2020). The question then remains whether an emotional mediation account (using the GEMIAC instrument) has more explanatory power than the semantic differential technique (with its core dimensions of valence and arousal) when induced or felt (as opposed to perceived or associated) emotions are concerned.

#### 1.1.2. Emotions evoked by visual shapes

Reliable associations between perceived emotions and visual shapes have been documented in a number of studies, where shapes were either selected from a repertory or freely drawn (Poffenberger and Barrows, 1924; Karwoski et al., 1942; Lyman, 1979; Collier, 1996). Three of the most studied qualitative properties of visual shape, in relation with their emotional effects, are curvature, symmetry, and complexity. Visual shape curvature or roundness has been extensively assessed in connection with the “curvature effect,” the preference for curved over sharp-angled contours (for a review, see Corradi and Munar, 2020). Vartanian et al. (2013) combine behavioral and neural evidence to show that this effect is probably driven by pleasantness. With respect to emotional arousal induced by visual contours, Blazhenkova and Kumar (2018) found curved and angular shapes to be associated with relieved and excited/surprised emotions, respectively.

Visual symmetry is a property traditionally associated with beauty and aesthetic preference (Weyl, 2015; Weichselbaum et al., 2018; also cf. Leder et al., 2019). Bertamini et al. (2013) reported that aesthetic responses to visual patterns with reflectional symmetry involved both positive valence and high arousal. Salgado-Montejo et al. (2015) also demonstrated preferential matching of symmetry (asymmetry) of visual shapes with the word “pleasant” (“unpleasant”). Pleasantness has also been shown to mediate associations between the (a)symmetry of visual shapes and taste (Turoman et al., 2018).

Ratings of visual shape complexity have been shown to depend on diverse sources as well, such as the number of sides or turns in the shape, asymmetry, compactness, degree of self-similarity, or shape skeletons based on local axes of symmetry (Sun and Firestone, 2021). Physiological arousal has been shown to increase with complexity, while pleasantness has been found to approximate a Wundt curve or inverted “U” shape, that is, to increase with complexity up to a certain point but then decrease for highly complex shapes (Berlyne, 1970; Madan et al., 2018).

#### 1.1.3. Emotions evoked by materials

Compared to music and other visual features (e.g., shapes), materials seem to have a weaker capacity to evoke emotions (Crippa et al., 2012), with a tendency to neutrality with respect to valence (Marschallek et al., 2021). Nevertheless, some studies on aesthetics of materials have considered their capacity to evoke emotions, as well as analyzing the associated set of elicited emotions. Crippa et al. (2012), for instance, asked individuals to assess the emotions elicited by 9 different materials. They found that, in general, emotions evoked by materials were rather weak, with the most frequent emotions reported being satisfaction, joy, fascination, surprise, dissatisfaction and boredom. For a study investigating the emotions associated with a range of different materials (cotton, satin, tinfoil, sandpaper, and abrasive sponge), see Etzi et al. (2016).

#### 1.1.4. Crossmodal correspondences between sound/music and visual shapes/materials

When it comes to crossmodal correspondences between sound/music and visual shapes/materials, previous studies have shown that pitch height is consistently associated with visual shape (for a summary, see Küssner, 2017). Lower pitch tends to be congruently framed with curvy shapes, while higher pitch is matched with sharper angular shapes (Melara and O'Brien, 1987). Parise and Spence (2012) also found that tones with a sinusoidal waveform were associated with a curvy shape, while tones with a square waveform were more associated with a jagged one (i.e., a kind of bouba-kiki effect; Reinoso Carvalho et al., 2017). In Fernay et al. (2012), a synesthete and 10 control participants were asked to draw a shape for different vowel sounds and to define two colors, together with a vertical and horizontal position for it. Control participants showed crossmodal correspondences agreeing with the synesthete’s perceptions, such that vertical position and color brightness increased as pitch increased (see also Salgado-Montejo et al., 2016 and Küssner et al., 2014, for pitch-space associations during free hand movement tasks in a two- and three-dimensional space, respectively). Interestingly, the speaker’s gender influenced the way participants set the size of the shape as well (i.e., larger shapes for a male voice). The influence of individuals’ background and training on forming crossmodal correspondences between music/sound and visual shapes is exemplified in Küssner (2013) and Küssner and Leech-Wilkinson (2014). For instance, it was shown that musically untrained participants produce more diverse visual shapes than musically trained participants when asked to draw visual representations of a series of sine tones and short musical excerpts. Notably, the most complex and asymmetrical visual shapes associated with the auditory stimuli were produced by a dancer without musical background (Küssner, 2013). Adeli et al. (2014) reported that softer musical timbres were associated with blue, green, or light gray rounded shapes, while harsher timbres were matched with red, yellow, or dark gray sharper angular shapes. In their study, timbres involving elements of both softness and harshness were associated with a mixture of the two kinds of visual shapes being analyzed.

Crossmodal correspondences between music and materials have been much less studied, which is surprising in view of the importance of materiality for instrumental timbre. In a recent study on the timbre semantics of Western orchestral instruments, Wallmark (2019) reported that the domain of musical timbre is often conceptualized by terms that are applied also to properties of materials (i.e., soft, dry, hard, metallic, smooth), as well as to visual shapes (i.e., smooth, round, open, sharp). In Murari et al. (2015), non-verbal sensory scales for qualitative music description are proposed. Among other scales, the authors used wood, polystyrene, and sandpaper samples of different roughness to represent the qualities of hard vs. soft and smooth vs. rough, finding that their participants were consistent in their ratings of musical excerpts with respect to smoothness/roughness (but not to softness/hardness).

### 1.2. Hypotheses

Based on the body of research reviewed above, we formulated the following hypotheses focusing on specific properties of crossmodal correspondences, as well as on the predictability^1^ of the GEMIAC emotions and core affect from the associated musical excerpts, visual shape features, and materials.

1. Common emotional associations with stimuli in different senses have often been shown to underlie crossmodal correspondences, particularly those involving music (emotionally-mediated correspondences: Spence, 2020). In other words, when music is associated with a non-auditory object or feature, both are often perceived to convey the same emotion, enabling one to infer musical emotions from those elicited by non-musical stimuli. While musical emotions have been shown to be predicted by perceptual musical features alone, such as pitch, tempo, mode, or dynamics (Lange and Frieler, 2018), we hypothesize that music-induced emotions may be recovered more efficiently from joint information on the musical excerpts, visual shapes, and materials that have been associated with them:

*H1*: Music-induced emotions can be predicted by the joint information of musical excerpt and features of visual shape and materials arising in crossmodal correspondences with the emotions induced by this excerpt.

1. Core affect is a universal and ubiquitous basic aspect of subjective emotional experience, capable of describing the affective connotations of percepts in different sensory modalities (Collier, 1996; Russell, 2003). However, the GEMIAC emotion pairs have been selected because of their specific relevance to describe musical emotions; in consequence, some of them, such as “joyful, wanting to dance” and “enchanted, in awe” might not be easily applicable to the visual shapes and usual design materials considered in this study. Moreover, the possibility of choosing between several similar GEMIAC emotions may diminish the predictive relevance of each individual one. Due to its universality, we hypothesize that we will obtain more accurate predictability of core affect than of discrete music-specific emotions such as those solely relying on GEMIAC:

*H2*: Valence and arousal of core affect induced by music will be predicted more accurately by the associated musical excerpts, visual shapes, and materials, compared to discrete GEMIAC emotions.

1. Materials seem to have a weaker capacity for evoking emotions, when compared to music, other visual features, and even other sensory modalities, such as taste and olfaction (i.e., Crippa et al., 2012). Emotions evoked by materials seem to be rather weak, with a tendency toward neutrality with respect to valence (Marschallek et al., 2021). Nevertheless, some studies on aesthetics of materials have considered their capacity to evoke emotions and to elicit crossmodal associations (Barbosa Escobar et al., 2023), as well as analyzing the associated set of elicited emotions. Some of these results suggest that materials may play a role for the emotional experience. However, and based on the above, we hypothesize that visual shapes are still better predictors of music-induced emotion than any specific material. We also hypothesize that a musical excerpt is an even better predictor of music-induced emotions, compared to visual shapes and materials crossmodally associated to these emotions:

*H3*: Materials by themselves will be poorer predictors of music-induced emotions and their valence and arousal than visual shapes. In turn, musical excerpts will be better predictors of music-induced emotions than visual shapes and materials.

## 2. Materials and methods

### 2.1. Participants

A total of 176 participants completed the experiment, 23 in English (12 female, 11 male) and 153 in Spanish (75 female, 77 male, 1 other), with a mean age of 33.20 years (SD = 11.02) and age range of 19–56 years. Participants resided in 10 countries (Germany, Argentina, Colombia, Australia, Italy, United Kingdom, United States, India, France, and Belgium) and were recruited by means of convenience sampling among the authors’ networks. None of the participants reported auditory or visual limitations. In order to assess the participants’ level of musical training/sophistication, we applied the single item measure described by Zhang and Schubert (2019). The resulting self-evaluation percentages were: nonmusicians, 19%; music-loving nonmusicians, 45%; amateur musicians, 18%; serious amateur musicians, 7%; semiprofessional musicians, 5%; and professional musicians, 6%. Thus, participants had a wide range of musical competence, with a majority of non-musicians. A participant information and consent form were built into the first page of the study, and all participants gave their informed consent before proceeding to the study itself.

### 2.2. Musical stimuli

Eight excerpts from the Eerola and Vuoskoski dataset of emotional film music (Eerola and Vuoskoski, 2011) were used as stimulus material. These excerpts have been consistently rated in valence and arousal and classified across discrete emotions *expressed* by the music and were shown to elicit a variety of induced emotions when modeled by the GEMS (Vuoskoski and Eerola, 2011), of which GEMIAC is an extension. Importantly, cluster analysis showed that low-level clusters of ratings of emotional excerpts were the same for the GEMS and discrete models (namely, four clusters corresponding respectively, in the discrete model, to Scary, Happy, Sad and Tender emotions; Vuoskoski and Eerola, 2011). Specifically, for our study, we selected two *Scary*, two *Happy*, two *Sad* and two *Tender* musical excerpts from those used in the aforementioned study: “Oliver Twist” and “Dances with Wolves” (named here *Happy 1* and *Happy 2*, respectively), “The English Patient” and “Running Scared” (*Sad 1* and *Sad 2*, respectively), “The Portrait of a Lady” and “Shine” (*Tender 1* and *Tender 2*, respectively), and “The Alien Trilogy” and “Batman Returns” (*Scary 1* and *Scary 2*, respectively). The duration of these musical excerpts varied between 46 and 72 s.

### 2.3. GEMIAC

The GEneva Music-Induced Affect Checklist (GEMIAC) comprises 14 classes of musical emotions, denoted by term pairs, such as “*melancholic*, *sad*” and “*moved, touched”* (Coutinho and Scherer, 2017). In the usage proposed by its authors, it is required to rate the experienced intensity of each emotion class after listening to a piece of music. Instead, here we asked participants to choose the better matching emotion class in response to each musical excerpt. We did so in order to later obtain a single representation of the (most characteristic) music-induced emotion for each excerpt, both as visual shape and material from a list (see Procedure). For the Spanish translation of the GEMIAC, we followed the methodology proposed by Vallerand (1989).

### 2.4. Procedure

The experiment was designed in Qualtrics and conducted online, either in English or in Spanish depending on the respondent’s language preferences. Informed consent was a prerequisite for taking part in the study. Participants were asked to use headphones at all times.

First, participants listened to a sample audio (not included in the set of stimuli) to adjust the sound volume to a comfortable level. Second, as the main task, the eight excerpts were presented in a randomized order. After listening to each excerpt, participants were asked to choose a single term pair from the GEMIAC scale to describe their induced emotion. The precise instruction for the participant was as follows: “*Please indicate which of the following term pairs best describes the emotion you experienced when listening to this audio. DO NOT DESCRIBE the music* (*Example: “this music is melancholic, sad”*) *or what the music seemed to express* (*Example: “this music expresses joy”*)*. Describe YOUR OWN EMOTION while listening to the music* (i.e.*, “I feel melancholic/sad while listening to this music”*)*. If you consider that your emotion does not correspond to any of the term pairs, choose from the list the term pair closest to the emotion you experienced*.”

Having selected a GEMIAC term, they were further asked to draw their emotion (“*Please draw a CLOSED SHAPE that represents THE EMOTION you experienced while listening to the music. You can erase the drawing as many times as you like*”). To record these shapes, we used the Signature feature provided by Qualtrics, which allows drawing using the mouse or touchpad of the computer.

As a final step, participants were asked to select a material that they thought would match the corresponding induced emotion. The list of materials, included in Table 1, was taken from Keyshot, a 3D rendering software used by designers (Jo, 2012). The instruction was: “*Please select from the given list of materials the material that you think best corresponds with the previously chosen emotion*.”

**Table 1:** Summary statistics of covariates.

Covariates		Code	N/mean	%/sd
Materials	Ceramic	X1	62	4.40%
	Glossy plastic	X2	9	0.64%
	Grained wood	X3	99	7.03%
	Granite	X4	89	6.32%
	Leather	X5	6	3.27%
	Fine grain wood	X6	55	3.91%
	Marble	X7	103	7.32%
	Nylon	X8	57	4.05%
	Opaque glass	X9	52	3.69%
	Opaque liquid	X10	68	4.83%
	Opaque metal	X11	97	6.89%
	Opaque plastic	X12	34	2.41%
	Porcelain	X13	87	6.18%
	Rubber	X14	46	3.27%
	Shiny metal	X15	119	8.45%
	Shiny plastic	X16	43	3.05%
	Transparent glass	X17	80	5.68%
	Transparent liquid	X18	89	6.32%
	Transparent plastic	X19	21	1.49%
	Transparent stone	X20	45	3.20%
	Velvet	X21	107	7.60%
Shapes	Complexity	C	20.01	11.99
	Roundness	R	40.83	12.00
	Symmetry	S	38.67	22.41

### 2.5. Shape features

The visual shapes reported by participants mentioned in the paragraph above were evaluated independently by two raters who did not participate in the study or its design. Using graphic sliders with a 0–100 range, they rated the three shape features described in Section 1.1.2. in scales for symmetry, either reflectional or rotational (from very asymmetrical to very symmetrical), roundness (from no roundness to high roundness), and complexity (from very simple to very complex). The raters had been previously instructed about 2D symmetry and the notion of curvature. Complexity was left as an intuitive notion.

### 2.6. Data

A total of *N* = 1,408 responses were gathered from 176 participants. Covariates include the musical excerpt, shape characteristics, and materials, though the interest resides in the latter two upon adjusting for the former. Shape characteristics were assessed based on symmetry, roundness, and complexity and mapped to a 0–100 scale, while the musical excerpt and materials were kept as categorical explanatory variables.

### 2.7. Statistical analysis

The software used for statistical analysis was R, version 4.2.1. Initially, we conducted an analysis of the predictive quality of the covariates separately for each GEMIAC emotion class. Covariate relevance was ranked using the Decreased Gini score metric (Breiman, 2001). Predictive quality was explored by calculating the area under the curve (AUC) for each GEMIAC class independently, and 95% confidence intervals for the AUC were also reported. Values above 0.80 are oftentimes considered to represent an excellent classifier (Hosmer et al., 2013), with an upper bound of 1 representing perfect classification. Several emotion classes stood out with high AUC: “tense, uneasy,” “powerful, strong” “energetic, lively” and “melancholic, sad.” However, other emotion pairs had relatively low AUC predictability.

**Figure 1:**
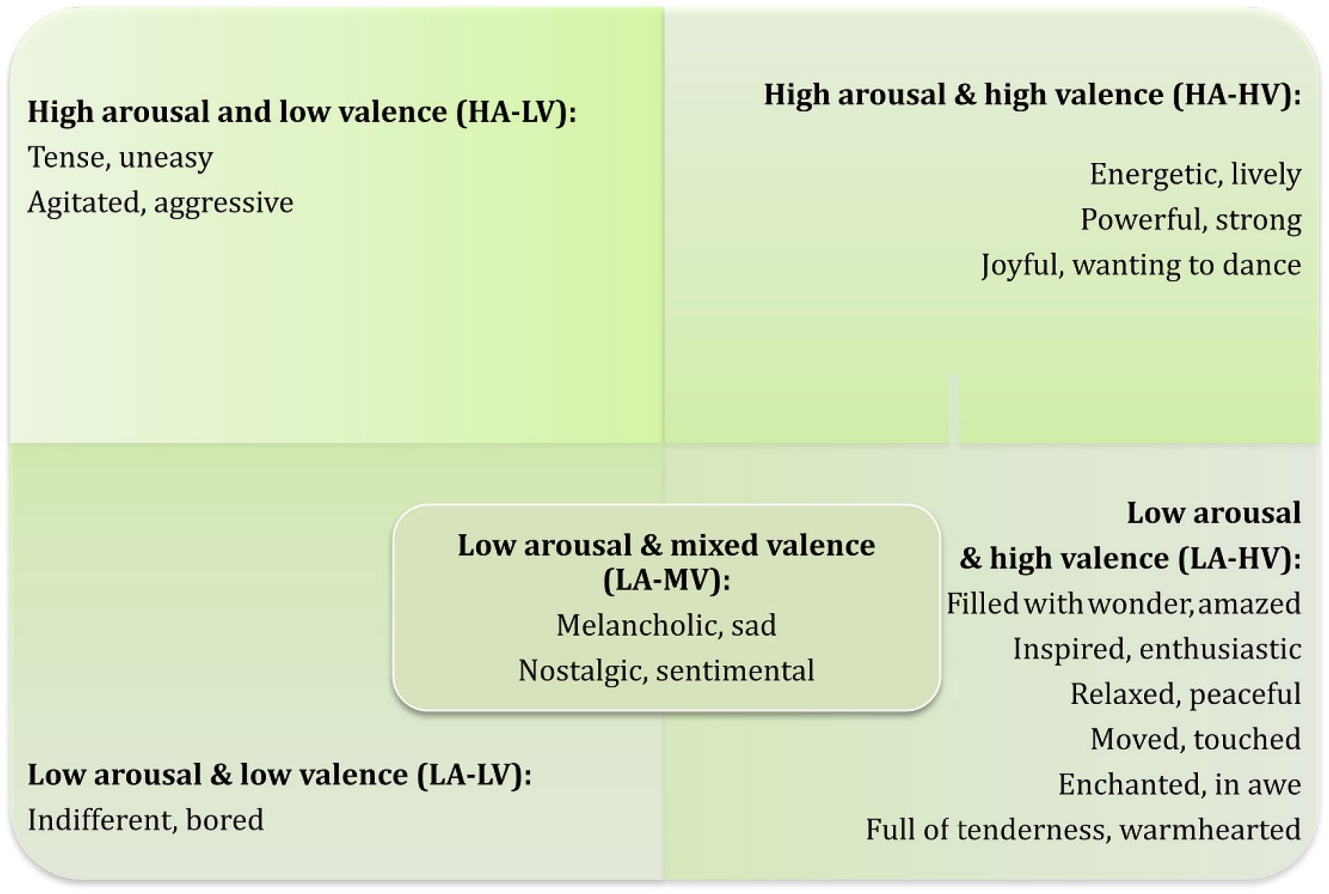
GEMIAC emotion terms grouped by arousal category (rows) and valence category (columns).

Therefore, emotions were grouped according to five relatively homogeneous valence-arousal pairs, as described in Figure 1, which form the categories within the primary analysis. These pairs represent the spectrum of emotions anticipated to be experienced by respondents, so that intra-group emotions share similarities (and represent common traits in the underlying emotions), but inter-group differences in emotion type are more clearly identifiable by the respondent (and represent more relevant differences in the underlying emotions).

Summary statistics were produced for the covariates as well as both the ungrouped and grouped emotions. A derivation cohort was defined using 75% of the data, and the remainder of the sample was used as a validation (out-of-sample) cohort. Since the associations between the covariates (musical excerpt, shapes, and materials) and the responses (pairs of valence-arousal emotions) were expected to have a complex and non-linear form, in line with the complexity associated with human information processing and decision-making, a machine learning-based method, random forest, was fit for the derivation cohort.^2^ The fitted random forest was subsequently used to demonstrate the joint out-of-sample predictive power of the covariates using solely the information in the validation cohort. In order to maintain full out-of-sample validity of the study, a participant effect was not included, though in practice the approach can be enhanced by including any known participant characteristics or prior responses if those are available prior to the experiment.

Predictive quality was explored by calculating the AUC for each valence-arousal pair independently, and 95% confidence intervals for the AUC were also reported. In order to understand whether core emotion features (arousal or valence) were independently associated with the covariates, two sensitivity analyses were performed: (1) collapsing valence across arousal categories, resulting in two broader categories for emotions (low & high arousal); and (2) collapsing arousal across valence categories, resulting in three broader categories for emotions (low, mixed, and high valence).

## 3. Results

**Figure 2:**
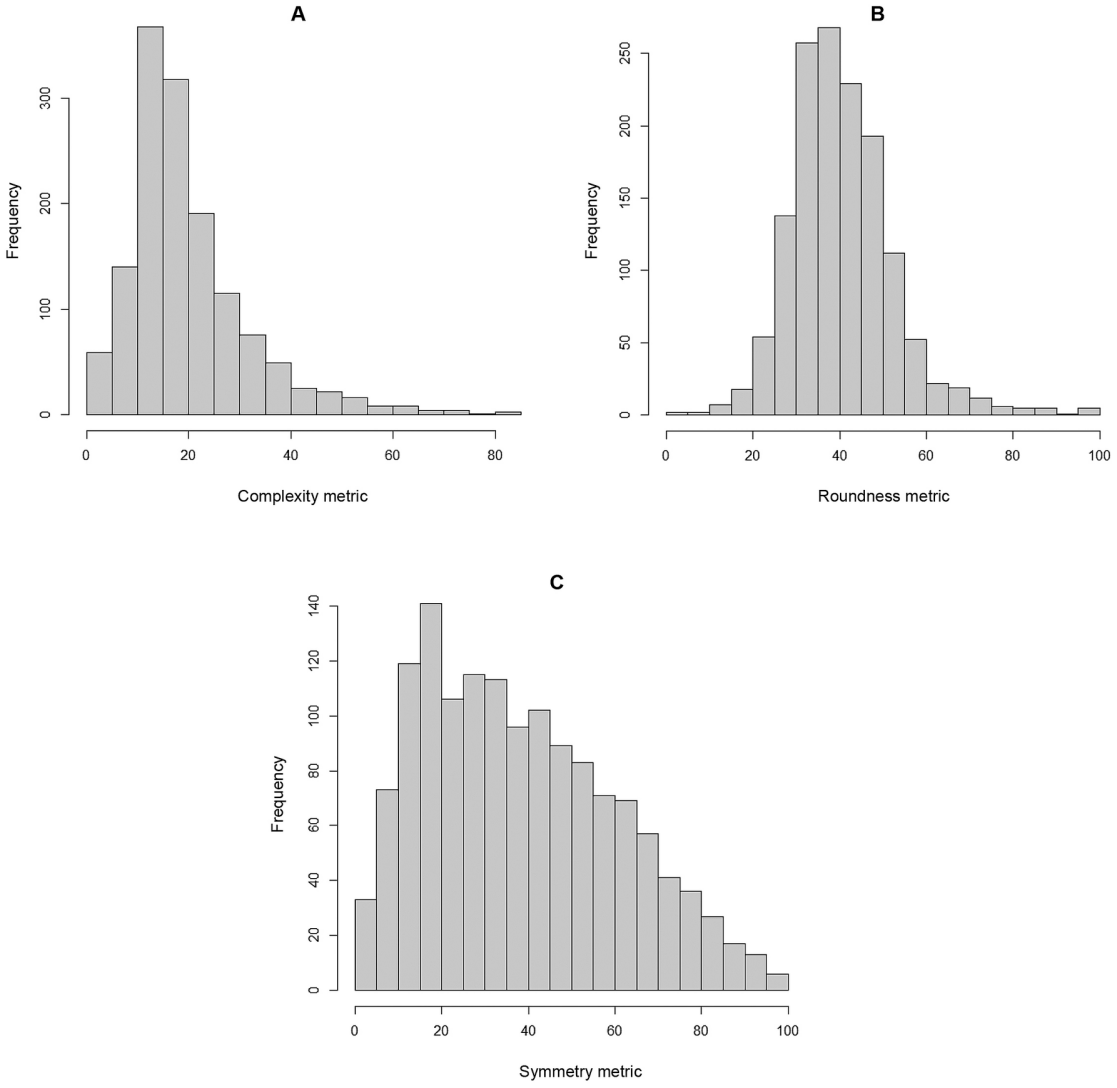
Distributions of the shape metrics **(A)** complexity, **(B)** roundness, and **(C)** symmetry across musical excerpts and participants.

Table 1 contains the summary statistics for the covariates, and Table 2 contains both the ungrouped and grouped emotions, where grouping of emotions is depicted in Figure 1. The majority of respondents selected low arousal (61.51%) and high valence (59.66%) emotions, with the combination of both categories representing the majority of responses (39.77%). The category with least responses corresponds to the combination of low valence and low arousal, which was selected by respondents in 4.29% of the experiments. Among materials, the most common ones were shiny metal (8.45%) and velvet (7.60%), while glossy plastic was the least common (0.64%). The metrics representing complexity and symmetry are right-skewed, while the distribution of roundness is more symmetric, as depicted in Figure 2. Complexity and symmetry were negatively correlated (r = −0.21; 95% CI –0.26, −0.16), while complexity and roundness were not found to be associated (r = 0.02; 95% CI –0.03, 0.07). Conversely, roundness and symmetry were found to be positively associated (*r* = 0.23; 95% CI 0.18, 0.28).

**Table 2:** Summary statistics of emotions: both ungrouped and grouped by valence and arousal category and further collapsed across valence and arousal categories.

Responses	*N*	%
Agitated, aggressive	50	3.55%
Enchanted, in awe	73	5.18%
Energetic, lively	107	7.60%
Filled with wonder, amazed	77	5.47%
Full of tenderness, warmhearted	46	3.27%
Indifferent, bored	59	4.19%
Inspired, enthusiastic	154	10.94%
Joyful, wanting to dance	49	3.48%
Melancholic, sad	122	8.66%
Moved, touched	96	6.82%
Nostalgic, sentimental	125	8.88%
Powerful, strong	124	8.81%
Relaxed, peaceful	114	8.10%
Tense, uneasy	212	15.06%
Low arousal, low valence (LA-LV)	59	4.19%
Low arousal, mixed valence (LA-MV)	247	17.54%
Low arousal, high valence (LA-HV)	560	39.73%
High arousal, low valence (HA-LV)	262	18.61%
High arousal, high valence (HA-HV)	280	19.89%
Low valence (LV)	321	22.80%
Mixed valence (MV)	247	17.54%
High valence (HV)	840	59.66%
Low arousal (LA)	866	61.51%
High arousal (HA)	542	38.49%

**Figure 3:**
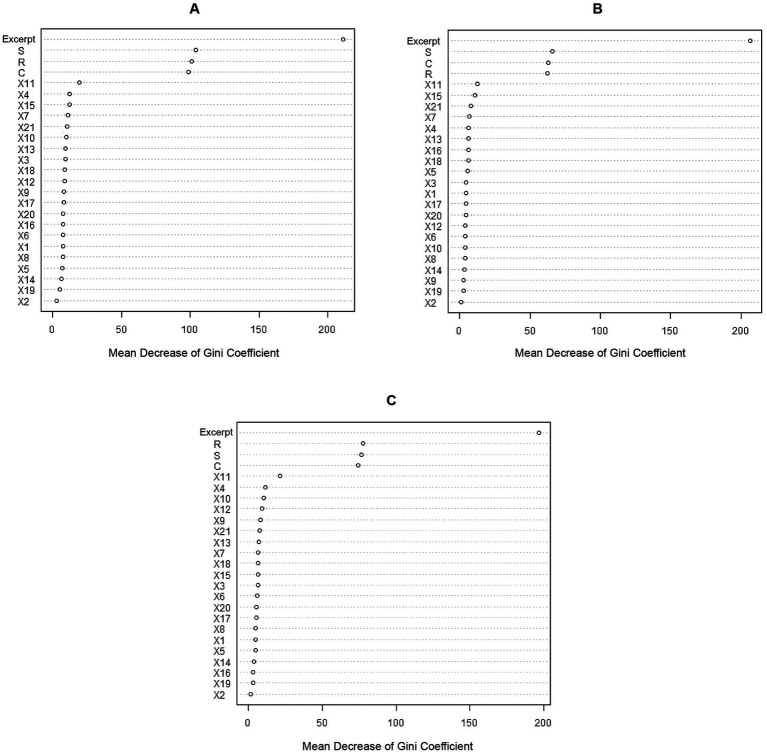
Mean decrease of Gini coefficient by covariate for the primary analysis **(A)**, and the two secondary analyses: **(B)** arousal grouped by valence and **(C)** valence grouped by arousal. C, complexity; R, roundness; S, symmetry. For all other codes (X1–X21), see Table 1.

Figure 3 includes a graphical representation of the random forest by mean decreased Gini scores in the derivation cohort for the overall analysis (by arousal-valence pair) as well as the two sensitivity analyses (grouping valence by arousal and arousal by valence). Variables are ranked by contribution to node homogeneity. Upon controlling for the musical excerpt, shape variables provided a higher contribution to enhanced node homogeneity than materials, which aligns with hypothesis H3.

**Figure 4:**
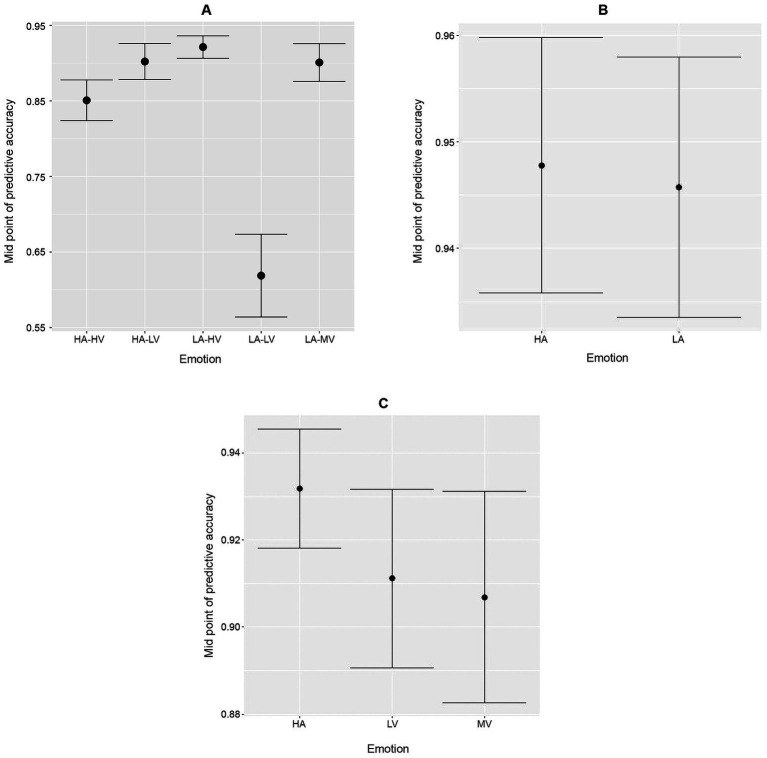
Out-of-sample AUC midpoint estimates and 95% confidence intervals for the validation cohort under the response categorizations in the primary analysis **(A)**, and the two secondary analyses: **(B)** arousal grouped by valence and **(C)** valence grouped by arousal. HA, high arousal; HV, high valence; LA, low arousal; LV, low valence; MV, mixed valence.

Figure 4 portrays the out-of-sample predictive/classification power (AUC) for each emotion category across the primary and sensitivity analyses for the validation cohort. Our results demonstrate a high level of predictive power across all emotions except for the low arousal-low valence combination, as seen in the left panel of Figure 4. This category could represent a basket choice of very heterogeneous emotions, such as true low arousal-low valence emotions or simply exhaustion and lack of interest in the experiment from participants. Also, this category contains the fewest number of data points (59 responses across both validation and derivation datasets, or 4.19%), which affects the ability of the random forest algorithm to learn efficiently about this category. For all other categories in the primary analysis, the approach demonstrates exceptional out-of-sample predictive power within the validation cohort, with AUC values above 0.85. When collapsing by arousal or valence, the predictive power across all categories remains excellent, with all AUC point estimates between 0.89 and 0.95, as demonstrated in the middle and right panels of Figure 4. This indicates that levels of both arousal and valence can be identified independently and jointly.

**Figure 5:**
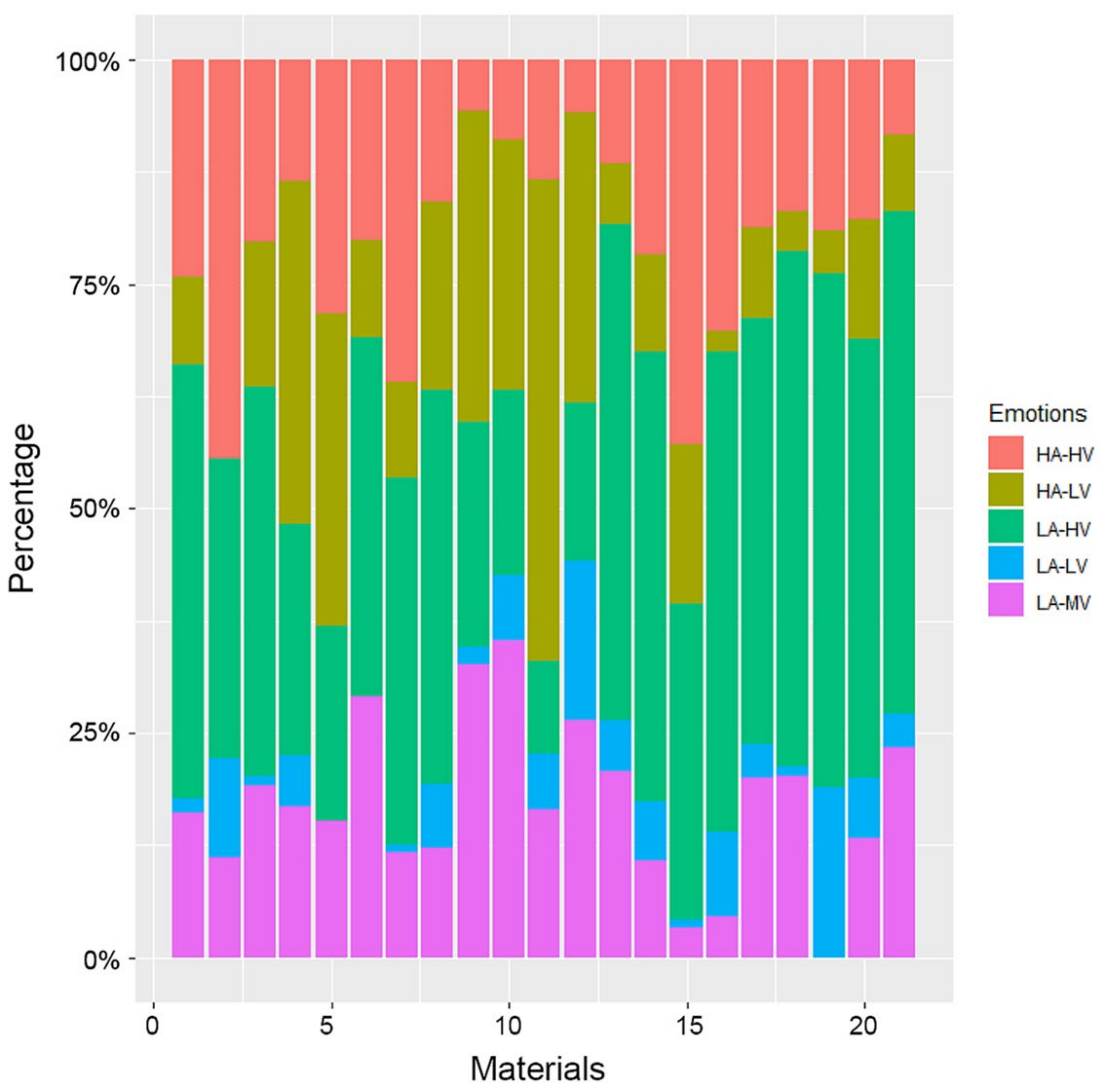
Observed proportions of emotion by material type (categorized as per Table 1). HA, high arousal; HV, high valence; LA, low arousal; LV, low valence; MV, mixed valence.

**Figure 6:**
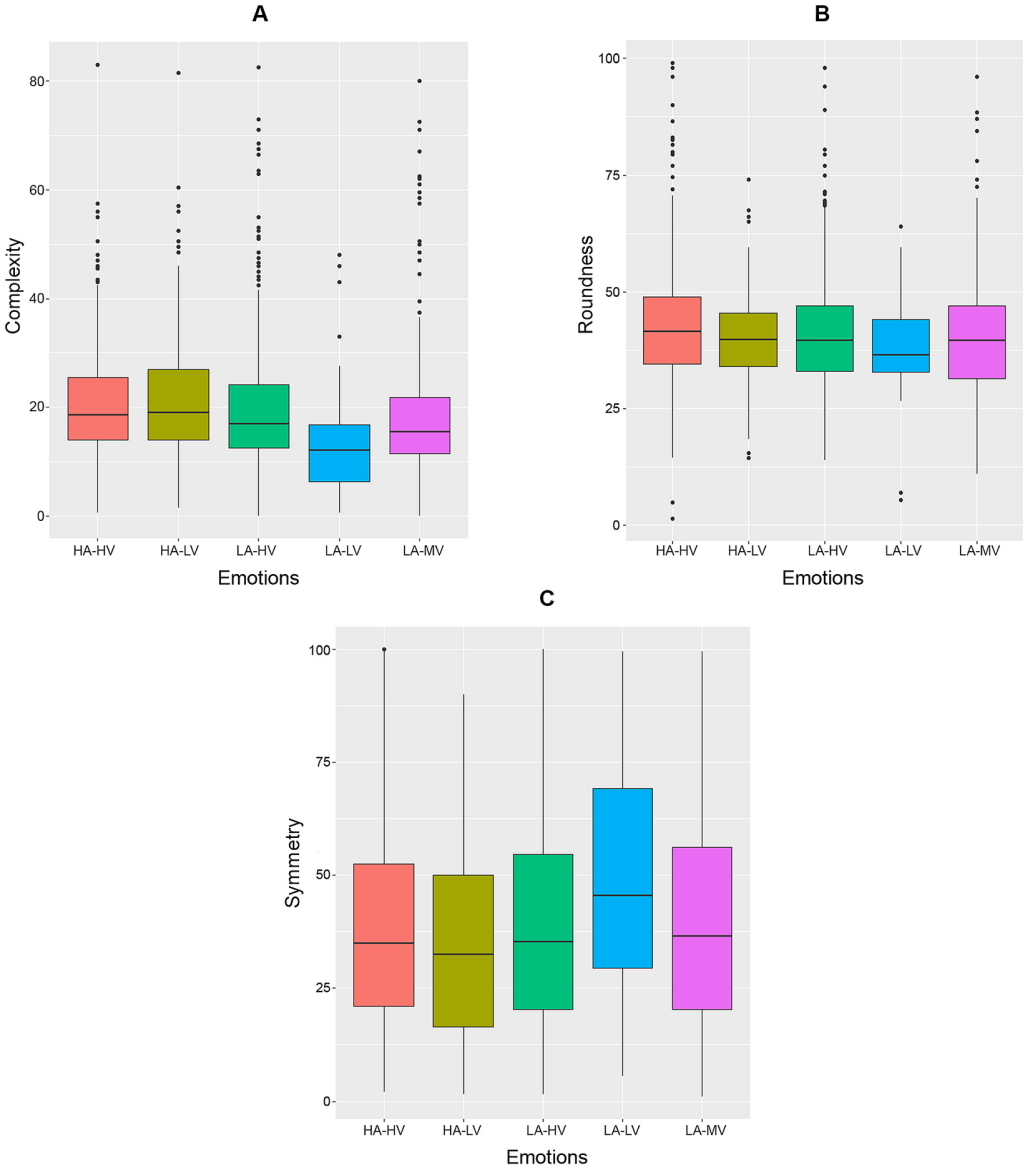
Boxplots of **(A)** complexity, **(B)** roundness, and **(C)** symmetry by emotion. HA, high arousal; HV, high valence; LA, low arousal; LV, low valence; MV, mixed valence.

In Figure 5, the associations between the covariates and the outcome variable from the primary analysis are (univariately) descriptively visualized. For example, opaque glass (Material 9 as per the ordering in Table 1) was associated with a lower observed frequency of high arousal, high valence emotions than materials such as glossy plastic (Material 2) or shiny metal (Material 15). Similarly, low arousal and low valence were negligible for shiny metal (Material 15), while they were observed in higher proportion for transparent plastic (Material 19). When grouping observations across valence (with HA-HV plus HA-LV constituting the high arousal broader category), we observed that high arousal is largest among observations of opaque metal and leather (Materials 11 and 5, respectively), while low arousal categories prevail among observations of velvet and porcelain (Materials 21 and 13, respectively). Figure 6A demonstrates, for example, how low arousal categories are associated with smaller levels of complexity than high arousal categories.

## 4. Discussion

The observed crossmodal correspondences between music-induced emotions and visual shapes/materials demonstrate some level of heterogeneity in emotional response univariately by covariate (Figures 5, 6). Consistently with previous findings, we observed an increase of arousal with shape complexity (Figure 6A), whereas our results on associations between materials and emotions appear to be new. The strong explanatory power demonstrated in Figure 4 comes from more complex, non-linear multivariate associations between the covariates, such as those extracted by the random forest approach. This indicates that emotional responses cannot be simply explained by compounding one-dimensional associations, and a more complex structure is needed, in line with how individuals process information. This predictive power is in agreement with our hypothesis H1.

We obtained further evidence for hypothesis H1 in the predictive analysis of the full 14 GEMIAC emotion pairs. Some of these emotions showed high AUC: “tense, uneasy” (AUC = 0.85), “powerful, strong” (AUC = 0.74), “energetic, lively” (AUC = 0.78) and “melancholic, sad” (AUC = 0.79). As such, we obtained good predictability for some highly specific musical emotion classes (Palmer et al., 2016). It could be that the names of these classes have more multimodal applicability, for instance, “sad” can be applied to shapes (Sievers et al., 2019), music, and materials (Zuo et al., 2001).

However, overall, the mixed performance of the random forest algorithm for music-specific emotions is in agreement with hypothesis H2, that is, there was less effectiveness in predicting specific GEMIAC emotion classes compared to core affect. This may have been due in part to the similarity of some of those emotion classes, such as “enchanted, in awe” (AUC = 0.56) and “filled with wonder, amazed” (AUC = 0.61), or to their low representativity for our excerpts, such as “full of tenderness, warmhearted,” amounting to only 3.27% of the responses (AUC = 0.59). These results can be compared to those of Meuleman and Scherer (2013), where predictive accuracy decreased as the number of clusters of emotions increased from two (positive vs. negative emotions) to twelve. Moreover, our results can be linked to the traditional Osgood semantic differential technique (SDT) which aims to measure connotative meanings of (aesthetic) objects, concepts or events (Osgood et al., 1957). While empirical studies of music in the tradition of Osgood’s SDT approach deal with associative, connotative meanings of music (for overviews, see Schubert and Fabian, 2006 and Spence, 2020), here we show that emotions *felt* by the listener can be predicted better when they are mapped onto core dimensions of SDT (i.e., valence and arousal) compared to the specific GEMIAC emotion classes.

Interestingly, in order to obtain this level of prediction efficiency for music-induced core affect, it was important to categorize the terms “melancholic, sad” and “nostalgic, sentimental”—which would seem to be low valence, low arousal (LV-LA) emotions—in a different valence-arousal group. These emotions—sometimes framed as “negative”—have been considered, nonetheless, as emotions that make a positive contribution to aesthetic liking. People may enjoy feelings of nostalgia and melancholy when listening to sad music, which can evoke not only sadness, but also a wide range of complex and partially positive emotions, such as peacefulness, tenderness, transcendence, and wonder (Taruffi and Koelsch, 2014). In particular, nostalgia has been conceptualized variously as a negative, ambivalent, or positive emotion (Sedikides et al., 2004). Consequently, we have categorized these terms in a separate mixed valence, low arousal (MV-LA) group. In contrast with its excellent performance when we take this group into account, running the random forest algorithm instead with “melancholic, sad” and “nostalgic, sentimental” grouped as LV-LA (so considering 4 emotion clusters instead of 5), we obtained that these emotions were predicted worse upon grouping them when compared to predicting each individual one. In fact, with this 4-class grouping, LV-LA emotions had AUC = 0.56, while in the full GEMIAC analysis “melancholic, sad” had AUC = 0.79 and “nostalgic, sentimental” had AUC = 0.67. We noted also that HV-HA, HV-LA and LV-HA emotions were predicted worse in the 4-class than in the 5-class grouping, having AUC = 0.62, 0.62, and 0.78, respectively, in the former case.

In accordance with our hypothesis H3, materials by themselves were less relevant than visual shapes for predicting core affect (Figure 3), and the musical excerpts were the most relevant predictors. This lower predictive power of materials can be partially attributed to the diversity of options presented. We also expected this on the basis of the relative emotional neutrality of materials (Marschallek et al., 2021), which would prevent them from capturing emotional connotations. However, despite this supposed neutrality, we observed distinctive, nonrandom distributions of core affect associated with each material (Figure 5). Interestingly, in a study comparing prediction of discrete music-induced emotions using perceptual musical features (such as pitch, tempo, mode, dynamics) vs. crossmodal associations (warm, cold, rough, smooth, dark, bright), models based on crossmodal features (tactile and visual) performed better than those based on perceptual features in four out of six emotions, suggesting that music-induced emotions may be captured more clearly and directly by extra-musical characteristics than by music-specific dimensions (Lange and Frieler, 2018).

Our results aligned with previous similar findings using random forests classifiers. In fact, these classifiers have been shown to improve predictive accuracy of emotions, with respect to other linear and nonlinear methods, in different contexts, involving both music-specific and more general emotions. For instance, Meuleman and Scherer (2013) found random forests to have the best performance, among 14 linear and nonlinear models, for predicting event-related emotions from appraisal criteria of events such as relevance, consequences, causes, or coping. Vempala and Russo (2018) studied predictability of musical emotion judgments from audio features and physiological signals with machine learning methods, finding that linear and nonlinear methods achieved similar prediction performance from audio features, while more flexible nonlinear models such as neural networks or random forests were needed to capture the predictive capacity of physiology features.

## 5. Limitations, applications, and future work

Some limitations need to be considered regarding the methodology and generalizability of our findings. For instance, we explicitly asked our participants to wear headphones at all times (see Section 2.4.). Since this was an online survey, we could not ensure they complied with this instruction. We also decided to allow our participants to start developing their associations related to the emotions induced by the music, first, via the selection of a GEMIAC term and second, via a drawing, while leaving the material association at the very end. We adopted such an order because materials are poor predictors of music-induced emotions (see H3). Nevertheless, a balanced order of these tasks could be explored in future research. Also, to obtain a single response in terms of materials and visual shapes, we asked participants to select the emotion they experienced by choosing a single item from the GEMIAC, while it is possible that they may have felt several ones, in different intensities. Moreover, the graphical interface to draw the visual shapes is designed for signatures and does not seem to allow a flexibility and ease of input equivalent to hand drawing. However, none of the participants complained or reported any issues using it.

There has been little research on the impact of the visual environment and its materiality on musical emotions. The rare existing work has shown effects of videos on the emotional appraisal of music, as well as on the perception of musical features such as tempo and loudness (Boltz et al., 2009; Boltz, 2013). Another recent example is the study of Hauck et al. (2022) in which musical emotion ratings have been shown to be shifted by lighting conditions such as hue, brightness, and saturation. For example, a musical piece paired with red light was rated as more powerful than the same musical piece paired with green or blue light. In the same vein, given the crossmodal associations and predictability between visual shapes, materials, and musical emotions shown in our study, we would predict that emotions induced by a given musical piece are moderated by different environments, e.g., shown as projected images or in virtual reality, exhibiting various characteristics of materiality and visual shape design.

Thus, our findings suggest that visual shapes and materiality may be important factors to consider in sensory interactions where emotional synergy is sought between environment and music, for instance in hospitals, shopping centers, theaters and opera houses, art installations, and music therapy environments. In the field of art and aesthetics, our research may be extended to the analysis of works of visual music such as those of Oskar Fischinger and Norman McLaren (Gibson, 2023), as well as to the design of systems for visualizing music-induced emotions. Other applications may arise in sensory interactions design and multisensory experiences. For instance, in 2020, a scented visual installation entitled Emotional Plateware (Mesz and Tedesco, 2021) introduced digital conceptual designs of tableware intended to augment gastronomic experiences from a multisensory perspective. This installation was conceived from the results of a study on crossmodal correspondences of visual shapes, colors, smells, and materials with four emotions induced by music, which inspired both the shapes and materiality of the plateware and the visuals displayed in digital tablets embedded in it. Further applications may arise in the field of sensory marketing as well. For instance, it is well-known that store atmospherics affect consumer behavior (Spence et al., 2014). In the constant look for novelty, as well as more engagement from consumers, brands may rely on correspondences such as the ones assessed in this study (i.e., music, shapes, and materials) in order to augment the user’s experience in retail from a multisensory perspective, either physically or digitally (Petit et al., 2019). Think of store atmospherics designed to evoke certain sensations and/or emotions congruently across music, visual shapes, and wall materials. We can also think of specific industries, such as fashion, where visual shapes and materials can be congruently associated with certain music in order to evoke certain emotions during the experience of consumers. Likewise, when thinking about the customer journey (e.g., Lemon and Verhoef, 2016), offline/online touch-point interactions may be reframed inspired by correspondences such as the ones being analyzed in this, and similar, work.

In conclusion, our results show the emergence of crossmodal correspondences between music-induced emotions and visual shapes and design materials, complementing previous findings on crossmodal correspondences between sound/music and their extra-musical elements (Küssner, 2013, 2017; Murari et al., 2015). We also show that visual shapes and materials capture and predict music-induced emotions, suggesting the possibility of designing congruent multimodal objects, environments, or messages oriented toward specific emotions, in spite of the interindividual diversity of “translations” across the senses (Spence, 2022). Future work should explore multisensory emotional design, combining music, visuals and materials.

## Data availability statement

The raw data supporting the conclusions of this article will be made available by the authors, without undue reservation.

## Ethics statement

The studies involving humans were approved by the Research Ethics Committee of Universidad Nacional de Tres de Febrero. The studies were conducted in accordance with the local legislation and institutional requirements. The participants provided their written informed consent to participate in this study.

## Author contributions

BM, ST, and MK conceptualized the study. BM, ST, MK, and FR-C developed the methodology and experimental protocol. BM and FR-C supervised the data collection. EH, GM, and LG analyzed the data and provided a report of the results. BM wrote the first draft of the manuscript. All authors revised and agreed on the final version of the manuscript.

## Funding

The article processing charge was funded by the Deutsche Forschungsgemeinschaft (DFG, German Research Foundation) – 491192747 and the Open Access Publication Fund of Humboldt-Universität zu Berlin. We also gratefully acknowledge the financial support provided by Universidad Nacional de Tres de Febrero.

## Conflict of interest

Author GM was employed by company Bayesian Solutions LLC, Charlotte, NC, United States.

The remaining authors declare that the research was conducted in the absence of any commercial or financial relationships that could be construed as a potential conflict of interest.

## Publisher’s note

All claims expressed in this article are solely those of the authors and do not necessarily represent those of their affiliated organizations, or those of the publisher, the editors and the reviewers. Any product that may be evaluated in this article, or claim that may be made by its manufacturer, is not guaranteed or endorsed by the publisher.

## Footnotes

^1^Predictability relates to the ability to use the information contained in the explanatory variables in order to infer what the corresponding emotions would be (future experiments) or have been (past experiments where the individual may not have disclosed the emotion). The hypothesis is that these associations between the covariates and the emotions are sufficiently consistent within and between individuals so that emotions are predictable if only the covariates are observed. However, such associations can potentially be substantially complex and non-linear, where covariates combine with each other and with themselves in complex ways, so a complex machine learning algorithm is needed to mimic how the brain operates linking those covariates in complex forms with the associated emotions.

^2^For another innovative analysis approach using non-linear models (a Gaussian process regression model with a linear plus squared-exponential covariance function) to study crossmodal associations between music/sound and visual shapes, see Noyce et al. (2013).
